# Glaucoma-Induced Optic Disc Morphometric Changes and Glaucoma Diagnostic Ability of Heidelberg Retina Tomograph II in Highly Myopic Eyes

**DOI:** 10.1371/journal.pone.0086417

**Published:** 2014-01-27

**Authors:** Chihiro Mayama, Tae Tsutsumi, Hitomi Saito, Ryo Asaoka, Atsuo Tomidokoro, Aiko Iwase, Shinichiro Otani, Kazunori Miyata, Makoto Araie

**Affiliations:** 1 Department of Ophthalmology, the University of Tokyo Graduate School of Medicine, Tokyo, Japan; 2 Department of Ophthalmology, Tajimi Municipal Hospital, Tajimi, Japan; 3 Miyata Eye Hospital, Miyakonojo, Miyazaki, Japan; 4 Kanto Central Hospital of The Mutual Aid Association of Public School Teachers, Tokyo, Japan; Medical University Graz, Austria

## Abstract

**Purpose:**

This study was performed to first investigate the morphological differences in the optic nerve head between highly myopic non-glaucomatous controls and highly myopic glaucomatous eyes in comparison with the differences between emmetropic non-glaucomatous controls and emmetropic glaucomatous eyes using confocal scanning laser ophthalmoscopy. Further, the ability of the apparatus in glaucoma diagnosis in highly myopic eyes was compared with that in emmetropic eyes.

**Methods:**

Healthy subjects and age-matched patients with early-stage open-angle glaucoma were divided into two groups: emmetropic eyes (−1.0 to +1.0 diopters) and highly myopic eyes (−12.0 to −5.0 diopters).The participants were comprised of 65 emmetropic normal eyes, 59 emmetropic glaucomatous eyes, 62 highly myopic normal eyes, and 68 highly myopic glaucomatous eyes and eyes with pathologic myopia were carefully excluded. Confocal scanning laser tomographic parameters were compared among all subjects after adjustment for age and disc area. The ROC curves and sensitivity and specificity for glaucoma detection using several clinical methods were then compared between the emmetropic and highly myopic eyes.

**Results:**

Rim area, cup/disc area ratio, mean cup depth, and cup shape measure of glaucoma eyes are significantly different from those of normal eyes in both highly myopic eyes and emmetropic eyes. Methodological overestimation of retinal nerve fiber layer cross sectional area due to optic disc tilting was suggested in the highly myopic eyes. The diagnostic performance of glaucoma using several discriminant methods significantly deteriorated in the highly myopic eyes.

**Conclusions:**

In the highly myopic glaucomatous eyes, confocal scanning laser tomographic parameters were significantly different from that of non-glaucomatous highly myopic eyes but diagnostic performance of glaucoma was deteriorated than that in emmetropic eyes. These findings demonstrate the utility and limitations of the apparatus in diagnosing glaucoma in highly myopic patients.

## Introduction

Morphological analysis of the optic nerve head (ONH) shape using imaging techniques has progressed in glaucoma diagnosis with obvious advantages in quantification and objectivity. Confocal scanning laser ophthalmoscopy (Heidelberg Retina Tomograph, Heidelberg Engineering GmbH, Heidelberg, Germany) is one of the most widely used and reliable methods for imaging ONH morphology. It reportedly provides satisfactory results in screening for glaucomatous eyes. [1−6].

Myopia is a common ocular pathology especially in Asians or in patients of Asian origin, [7] including in Japan.[8], [9] Myopia is correlated with a higher prevalence of open-angle glaucoma (OAG) in several epidemiological studies. [10−14] Tilting of the ONH and/or thinning of retinal nerve fiber layers (RNFL) is associated with myopia, [15], [16] which causes difficulty in detecting early glaucomatous changes in ONH morphology or RNFL defects, when using funduscopy or fundus photographs, especially in eyes with high myopia. Optic discs of highly myopic eyes are apparently different from those of emmetropic eyes, both morphologically and histologically. [17], [18] This causes more problems in glaucoma diagnosis when using imaging devices, including confocal scanning laser ophthalmoscopy or optical coherence tomography (OCT).

However, the use of confocal scanning laser ophthalmoscopy in detecting glaucoma in subjects with high myopia has rarely been reported because these eyes have been excluded from most clinical studies. There are only limited studies describing the features of the optic disc or RNFL in highly myopic eyes without glaucoma, using confocal scanning laser ophthalmoscopy [19] or OCT.[20], [21] Eyes with glaucoma were not included in these studies.

The purpose of this study is to compare the ONH morphology differences between highly myopic non-glaucomatous controls and highly myopic glaucomatous eyes with those of emmetropic non-glaucomatous controls and emmetropic glaucomatous eyes using confocal scanning laser ophthalmoscopy. Early-stage OAG patients with emmetropia or high myopia, and age matched non-glaucoma control groups with emmetropia or high myopia were recruited. Glaucoma diagnostic efficacy using confocal scanning laser ophthalmoscopy for highly myopic eyes was compared to emmetropic eyes using Frederick S. Mikelberg (FSM) discriminant function, [22] Reinhard O.W. Burk(RB) discriminant function(Heidelberg Retina Tomograph II Operating Instructions, Dossenheim, Germany), and Moorfields regression analysis (MRA). [3].

## Methods

### Subjects

Data from non-glaucoma subjects and OAG patients were acquired at three institutes in Japan, the University of Tokyo (Tokyo, Japan), Tajimi Municipal Hospital (Gifu, Japan), and Miyata Eye Hospital (Miyazaki, Japan) from the consecutive subjects visited either of the above institute between January 2007 and December 2008.The study protocol was approved by the institutional review board of each institution and adhered to the tenets of the Declaration of Helsinki. Written informed consent was obtained from each subject after explanation of the study protocol.

Self-reported healthy volunteers or subjects who visited the institutions for prescriptions for glasses, and were at least 20 years of age were invited to participate in the study (Table 1). The following ocular examinations were performed at the first visit: refraction and best-corrected visual acuity (BCVA) measurements using the 5-meter Landolt chart, slit-lamp examination, intraocular pressure (IOP) measurement using a Goldmann applanation tonometer, dilated funduscopy, and visual field testing using the Humphrey Field Analyzer 24-2 SITA standard program (HFA, Carl Zeiss Meditec, Inc., Dublin, CA). Those with spherical equivalent refractive errors of −1.0 to +1.0 diopter were assigned to an emmetropic group and those with −12.0 to −5.0 diopter were assigned to a highly myopic group.

**Table 1:** Demographic Data and HRT Parameters of the Subjects of Each Group in Global Analyses after Adjustment for Age and Disc Area.

Category	Demographic data				HRT parameters (global)						
	N (Male/female)	Age(year)	Refraction (diopter)	MD (dB)	Disc area (mm^2^)	Rim area (mm^2^)	Cup/disc area ratio	Mean cup depth (mm)	Height variation contour (mm)	Cup shape measure	RNFL cross sectional area (mm^2^)
Emmetropic normal eyes	65(31/34)	60.0±5.2	0.19±0.52	−0.49±1.1	2.00±0.34	1.55±0.29	0.22±0.11	0.18±0.07	0.37±0.09	−0.19±0.07	1.22±0.35
Emmetropic OAG eyes	59(12/47)	61.7±10.3	0.00±0.61	−4.0±2.0*	2.30±0.52*	1.08±0.27*	0.50±0.15*	0.33±0.11*	0.39±0.17	−0.08±0.08*	0.96±0.35*
Highly myopic normal eyes	62(29/33)	38.3±11.2	−7.6±1.8	−1.4±1.2	2.07±0.60	1.54±0.40	0.24±0.13	0.22±0.09	0.47±0.14	−0.17±0.07	1.54±0.46
Highly myopic OAG eyes	68(33/35)	41.0±6.4	−7.5±1.7	−4.2±2.3*	2.27±0.67	1.25±0.37*	0.42±0.17*	0.32±0.10*	0.53±0.18	−0.09±0.07*	1.59±0.62

N: number of eyes.

Refraction: spherical equivalent refractive error.

MD: mean deviation of Humphrey Field Analyzer 24-2 SITA standard program.

OAG: open-angle glaucoma.

* :*P*<0.05 with Bonferroni’s correction between normal and OAG eyes within emmetropic or myopic eyes.

(*P* values are adjusted for age and disc area, except that of age and disc area.).

Exclusion criteria were contraindication to pupil dilation, IOP of 22 mmHg or higher, BCVA worse than 0.7, unreliable results of HFA (fixation loss, false-positive, or false-negative >20%), glaucomatous visual field defects according to Anderson and Patella’s criteria as described below, [23] any abnormal visual field loss consistent with ocular disease, history of intraocular or refractive surgery, history of ocular or systemic diseases that could affect the results of confocal scanning laser ophthalmoscopy examinations, including clinically significant cataract, any retinal diseases including diabetic retinopathy/maculopathy or age-related macular degeneration, and optic nerve or RNFL abnormality. Eyes with signs suggestive of pathologic myopia were carefully excluded.

OAG patients from each institution fulfilling the following criteria were also included in this study. The inclusion criteria were: reproducible glaucomatous changes in the ONH with/without RNFL defect as confirmed by a glaucoma specialist (M.A., A.I., S.O.), glaucomatous visual field defects as shown by the HFA 24-2 SITA standard program obtained within 3 months of the Heidelberg Retina Tomograph (HRT) examination, mean deviation (MD) of the HFA 24-2 SITA standard program >−10 dB, and absence of history of other ocular pathological changes that could affect the results of HFA or HRT examinations, including intraocular surgeries or refractive surgeries. Glaucomatous visual field defects were defined according to Anderson and Patella’s criteria as follows: cluster of 3 or more points in the pattern deviation plot within a single hemifield (superior or inferior) with *P* values <5%, one of which must have a *P* value <1%. [23] Normal visual field was defined as not meeting the above criteria, glaucoma Hemifield Test results outside normal limits, and abnormal pattern standard deviation with *P*<5%. Patients with refractive errors of −1.0 to +1.0 diopter were assigned to an emmetropic group and those with −12.0 to −5.0 diopter were assigned to a highly myopic group, corresponding to the non-glaucoma subjects. Eyes with signs suggestive of pathologic myopia were carefully excluded.

If a non-glaucoma subject fulfilled the inclusion criteria bilaterally, one of both eyes randomly chosen was included, and if a non-glaucoma subject or an OAG subject fulfilled the inclusion criteria unilaterally, that eye was included in the study. If an OAG patient fulfilled the criteria bilaterally, an eye with worse MD of the HFA 24-2 program was included. Eyes with optic discs suggestive of anomaly not attributed to myopia were carefully excluded.

### HRT measurements

The ONH morphology was evaluated by confocal scanning laser tomography (Heidelberg Retina Tomograph [HRT] II; Heidelberg Engineering, Heidelberg, Germany; version 3.0 software) in each subject. HRT measurements were carried out as previously reported. [19] Five images were continuously taken, and mean topography images were calculated using three of the five images stored in each measurement. Standard deviations of the integrated images of less than 30 µm were used. A disk contour line was determined by an experienced operator (T. T.) for all images with reference to the fundus photographs taken simultaneously. The standard reference plane was 50 µm posterior to the mean height of the disk contour, located temporally between 350° and 356°.

Among the HRT parameters obtained, disc area, rim area, cup/disc area ratio, mean cup depth, height variation contour, cup shape measure, and RNFL cross sectional area were compared globally. Rim area, cup/disc area ratio, mean cup depth, and RNFL cross sectional area were also compared sector wise, which divided the disc into 6 sectors as follows (degrees in left eyes): temporal (316°–45°), temporal-superior (46°–90°), nasal-superior (91°–135°), nasal (136°–225°), nasal-inferior (226°–270°), and temporal-inferior (271°–315°).

The FSM discriminant function is automatically calculated by the apparatus using rim volume, height variation contour, cup shape measure, and age. [22] The RB discriminant function is also automatically calculated using height variation contour and cup shape measure. The eyes with measurements smaller than zero were diagnosed as glaucomatous. Results of MRA are calculated by the apparatus, using logarithm of rim area and disc area, and the eyes are identified as “outside normal limits”, “border line”, or “within normal limits”. Two definitions of glaucomatous eyes were adopted in this study. Only “outside normal limits” eyes were classified as being glaucomatous (MRA 1), and both “outside normal limits” and “border line” eyes as being glaucomatous (MRA 2). [3].

Receiver operating characteristic (ROC) curve and area under the curve (AUC) for each discriminant method were generated in both emmetropic eyes and myopic eyes using the values provided by Frederick S. Mikelberg (FSM) discriminant function, [22] Reinhard O.W. Burk(RB) discriminant function (Heidelberg Retina Tomograph II Operating Instructions, Dossenheim, Germany), and the calculated values based on the Moorfields regression analysis (MRA) formula. [3].

Sensitivity and specificity of HRT II for detecting glaucoma using FSM [22] and RB discriminant function and MRA [3] were also calculated in emmetropic and highly myopic eyes.

### Statistical analyses

HRT results were first compared between non-glaucoma subjects and OAG patients within the emmetropic or highly myopic eyes, respectively, by *t*-test with Bonferroni’s correction. Results from control subjects and OAG patients and from emmetropic and highly myopic eyes were also compared. In the analysis of HRT parameters, the effects of age and disc area were adjusted by analysis of covariance (ANCOVA) that included age and disc area as the covariance factors. Performance of HRT II for detecting glaucoma, using FSM, RB discriminant function, or MRA were compared by ROC curves. Statistical analyses were performed using a statistical software package, SPSS 15.0J for Windows (SPSS Japan Inc., Tokyo, Japan), and *P*<0.05 was adopted as a statistically significance level. The significance level was modified (with *P*<0.0125(0.05/4), *P*<0.007(0.05/7), or *P*<0.002 (0.05/24) with Bonferroni’s correction for demographic data of the subjects, global analyses, and sector wise analyses of HRT data, respectively.

## Results

Sixty-five emmetropic eyes of 65 non-glaucoma subjects, 59 emmetropic eyes of 59 OAG patients, 62 highly myopic eyes of 62 non-glaucoma subjects, and 68 highly myopic eyes of 68 OAG patients were enrolled in the study. Demographic data and obtained HRT parameters of the subjects are summarized in Table 1. Age showed no significant difference between control and glaucoma subjects within both emmetropic and highly myopic groups (*P*≥0.10). MD of HFA was −4.00±2.02 and −4.19±2.27 dB in the emmetropic and highly myopic OAG eyes, respectively (*P* = 0.62), and were lower than those obtained from the non-glaucoma eyes with corresponding refractions (*P*<0.001). The emmetropic subjects were older than myopic subjects in both non-glaucoma and OAG groups (*P*<0.001).

Global HRT parameters of the subjects are shown in Table 1. In emmetropic eyes, disc area was 2.00±0.34 mm^2^ in non-glaucoma eyes and 2.30±0.52 mm^2^ in OAG eyes with a significant difference between them (*P* = 0.0003). In the highly myopic eyes, disc area showed no significant difference between control and OAG eyes (*P* = 0.064). Rim area in OAG eyes was significantly smaller than in non-glaucoma eyes, both in emmetropic and highly myopic eyes (*P*<0.001). Cup/disc area ratio, mean cup depth, and cup shape measure were significantly greater in the OAG eyes than in the control eyes, both in the emmetropic and highly myopic eyes (*P*<0.001). Height variation contour was not significantly different between non-glaucoma and OAG eyes, both in the emmetropic and highly myopic eyes (*P*≥0.029). RNFL cross sectional area of OAG eyes was significantly smaller in the emmetropic eyes (*P*<0.001) but not in highly myopic eyes (*P* = 0.60).

The results of sector wise analyses are summarized in Table 2. In emmetropic eyes, there were significant differences in rim area, cup/disc area ratio, mean cup depth, and RNFL cross sectional area, between non-glaucoma and OAG eyes in all of the six sectors (*P*<0.0003).In the highly myopic eyes, rim area tended to be smaller in temporal-superior and nasal-inferior sectors though the differences were not significant after Bonferroni’s correction (*P*≥0.008), while cup/disc area ratio was significantly larger (*P*<0.001) in all of the sectors in OAG eyes compared with non-glaucoma eyes. Deepening in mean cup depth was not significant in temporal sectors after Bonferroni’s correction (*P* = 0.009), and RNFL cross sectional areas was not altered in OAG eyes except for temporal-inferior sectors (*P*<0.001).

**Table 2:** HRT Parameters of the Subjects of Each Group in Sector-wise analyses after Adjustment for Age and Disc Area.

Category	Rim area (mm^2^)	Cup/disc area ratio	Mean cup depth (mm)	RNFL cross sectional area (mm^2^)	Rim area (mm^2^)	Cup/disc area ratio	Mean cup depth (mm)	RNFL cross sectional area (mm^2^)	Rim area (mm^2^)	Cup/disc area ratio	Mean cup depth (mm)	RNFL cross sectional area (mm^2^)
	***Temporal sector***				***Temporal-superior sector***				***Temporal-inferior sector***			
Emmetropic normal eyes	0.25±0.06	0.49±0.14	0.26±0.09	0.12±0.03	0.21±0.04	0.24±0.15	0.26±0.12	0.22±0.06	0.21±0.05	0.25±0.15	0.22±0.10	0.20±0.05
Emmetropic OAG eyes	0.15±0.06*	0.70±0.15*	0.34±0.12*	0.09±0.03*	0.13±0.05*	0.55±0.21*	0.41±0.17*	0.16±0.06*	0.10±0.07*	0.69±0.21*	0.35±0.12*	0.08±0.10*
Highly myopic normal eyes	0.28±0.11	0.42±0.19	0.21±0.07	0.12±0.03	0.20±0.05	0.24±0.18	0.24±0.11	0.22±0.10	0.21±0.06	0.22±0.16	0.20±0.08	0.18±0.08
Highly myopic OAG eyes	0.22±0.08*	0.55±0.19*	0.25±0.07	0.11±0.04	0.17±0.06	0.45±0.23*	0.36±0.13*	0.25±0.13	0.14±0.08*	0.51±0.24*	0.28±0.08*	0.12±0.08*
	***Nasal sector***				***Nasal-superior sector***				***Nasal-inferior sector***			
Emmetropic normal eyes	0.47±0.10	0.09±0.09	0.14±0.09	0.41±0.13	0.24±0.05	0.12±0.12	0.18±0.11	0.26±0.07	0.25±0.04	0.09±0.09	0.15±0.09	0.26±0.06
Emmetropic OAG eyes	0.35±0.10*	0.34±0.22*	0.25±0.14*	0.26±0.14*	0.18±0.05*	0.36±0.20*	0.33±0.16*	0.21±0.07*	0.17±0.05*	0.39±0.19*	0.28±0.12*	0.16±0.08*
Highly myopic normal eyes	0.42±0.14	0.15±0.13	0.26±0.15	0.49±0.16	0.21±0.06	0.17±0.15	0.25±0.15	0.27±0.09	0.22±0.06	0.13±0.11	0.19±0.10	0.26±0.10
Highly myopic OAG eyes	0.34±0.13*	0.33±0.20*	0.38±0.17*	0.52±0.23	0.18±0.07*	0.37±0.21*	0.44±0.19*	0.32±0.14	0.21±0.07	0.31±0.18*	0.31±0.13*	0.28±0.13

OAG: open-angle glaucoma.

* : *P*<0.05 with Bonferroni’s correction between normal and OAG eyes within emmetropic or myopic eyes.

(*P* values are adjusted for age and disc area.).

**Figure 1:**
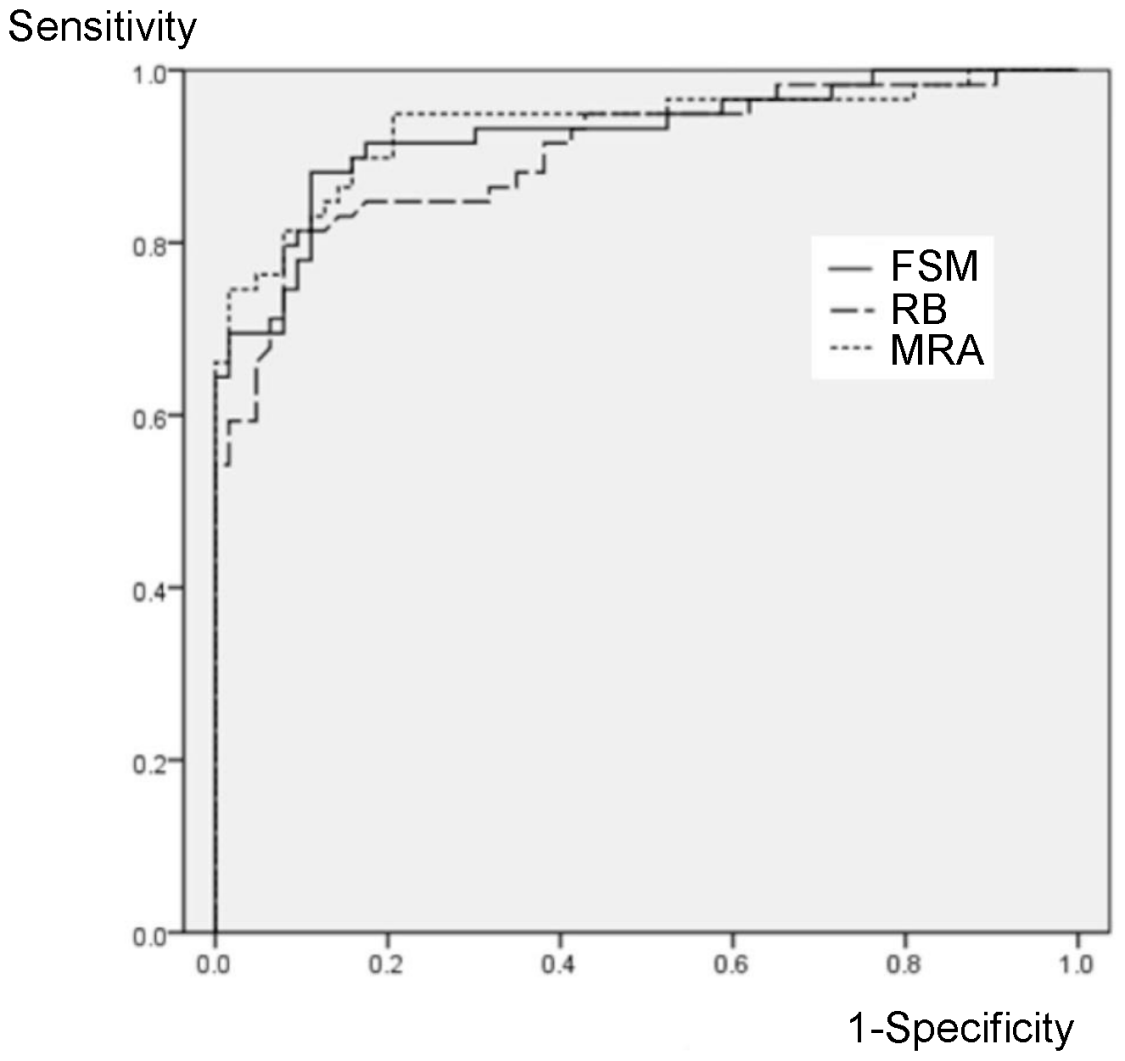
ROC curves for diagnosis of glaucoma in emmetropic eyes. AUCs of FSM or RB Discriminant Function and MRA are 0.926(95%CI: 0.878–0.974), 0.904(0.849–0.958), and 0.934(0.887–0.981), respectively. FSM: Frederick S. Mikelberg discriminant function; RB: Reinhard O.W. Burk discriminant function; MRA: Moorfields regression analysis.

**Figure 2:**
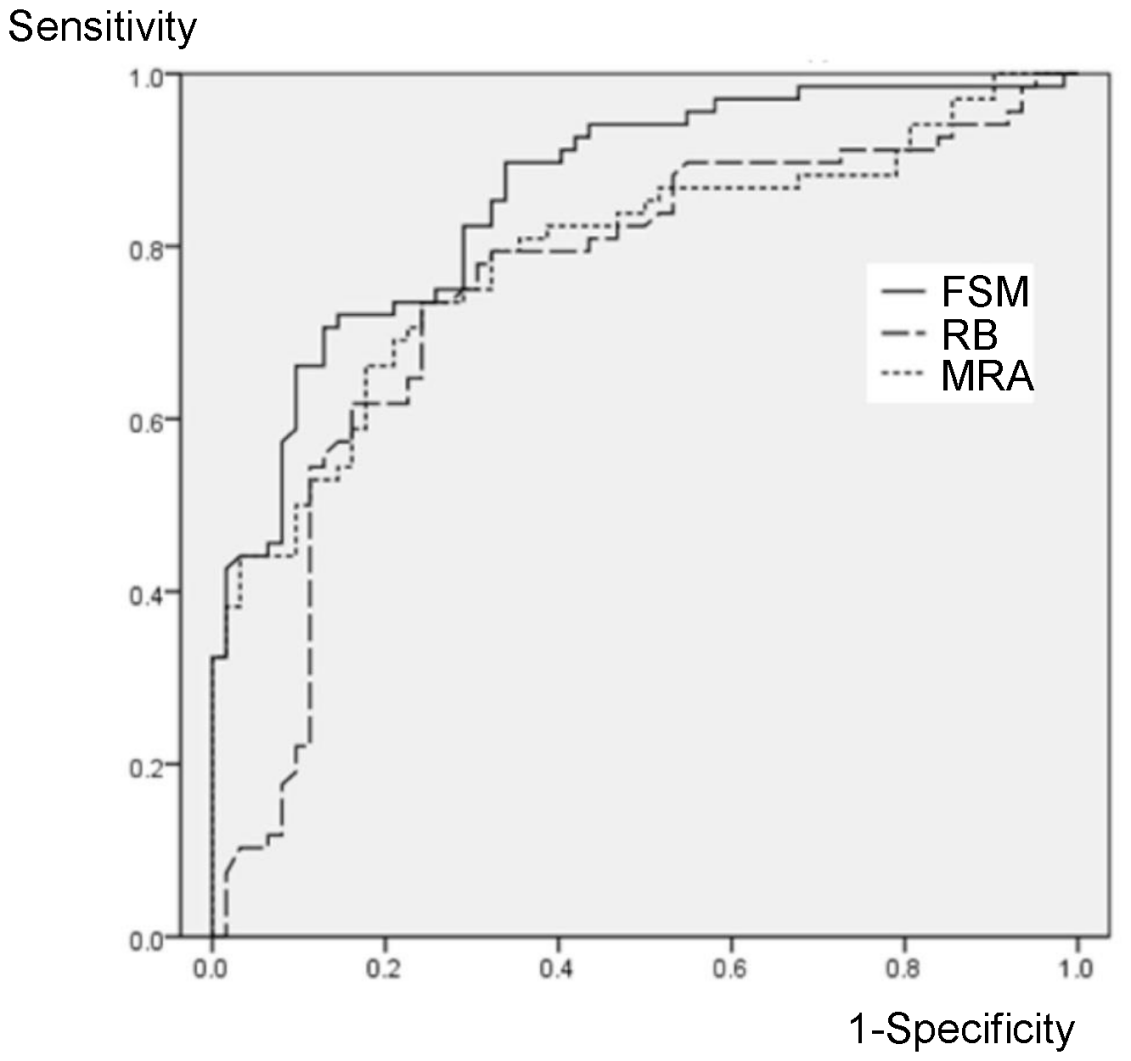
ROC curves for diagnosis of glaucoma in highly myopic eyes. AUCs of FSM or RB Discriminant Function and MRA are 0.859(95%CI: 0.797–0.922), 0.752(0.665–0.840), and 0.789(0.711–0.868), respectively. FSM: Frederick S. Mikelberg discriminant function; RB: Reinhard O.W. Burk discriminant function; MRA: Moorfields regression analysis.

ROC curves of FSM, RB discriminant function, and MRA for emmetropic and highly myopic eyes were shown in Figure 1 and 2, respectively. AUCs of the three methods were 0.926(95%CI: 0.878–0.974), 0.904(0.849–0.958), and 0.934(0.887–0.981) in emmetropic eyes without significant differences among them, while these were 0.859(0.797–0.922), 0.752(0.665–0.840), and 0.789(0.711–0.868), respectively, in the highly myopic eyes. AUC of FSM discriminant function was significantly larger than the others in the highly myopic eyes (*P* = 0.002 and 0.003). AUC of FSM discriminant function was not different between emmetropic and myopic eyes (*P* = 0.52), however, AUC of RB discriminant function and MRA was significantly lower in the highly myopic eyes than those in emmetropic eyes (*P* = 0.005 and 0.027, respectively).

Sensitivity and specificity of FSM, RB discriminant function, and MRA are shown in Table 3. Sensitivity and specificity were generally lower in the highly myopic eyes when compared to the emmetropic eyes. RB discriminant function and MRA 1 (only “outside normal limits” eyes were considered as being glaucomatous) had high specificity (1.00 and 0.98, respectively) of >0.95 in the emmetropic eyes, suggested as a clinically efficient level, [24] and both methods had relatively high specificity (0.90 and 0.89) in highly myopic eyes though sensitivity was low (0.22 and 0.51, respectively). FSM and MRA 2 (both “outside normal limits” and “border line” eyes were considered as being glaucomatous) showed moderate sensitivity and specificity in highly myopic eyes.

**Table 3:** Sensitivity and Specificity of FSM or RB Discriminant Function and Moorfields Regression Analysis.

Category		FSM	RB	MRA 1	MRA 2
Emmetropic eyes	Sensitivity	0.85	0.46	0.83	0.93
	Specificity	0.89	1.00	0.98	0.88
Highly myopic eyes	Sensitivity	0.75	0.22	0.51	0.76
	Specificity	0.71	0.90	0.89	0.74

FSM: Frederick S. Mikelberg discriminant function.

RB: Reinhard O.W. Burk discriminant function.

MRA 1: Moorfields regression analysis (only “outside normal limits” eyes were diagnosed as glaucomatous).

MRA 2: Moorfields regression analysis (“outside normal limits” and “border line” eyes were diagnosed as glaucomatous).

## Discussion

This study, for the first time, directly compared the HRT parameters between OAG eyes in early stage and age-matched controls in both emmetropic and highly myopic eyes. Considering the high prevalence of myopia in Asian populations, including Japan [7]–[9] and the correlation between myopia and OAG [10]–[14] diagnosis of glaucoma in highly myopic eyes is of major clinical importance.

In global analyses of HRT parameters, rim area, cup/disc area ratio, mean cup depth, and cup shape measure were significantly and similarly affected by glaucoma, both in emmetropic and highly myopic eyes. These results show that glaucomatous changes in the optic disc morphology are not substantially different between among emmetropic and highly myopic eyes, as far as the HRT findings were concerned. In the current study, height-variation contour showed no significant difference between non-glaucoma and OAG eyes both in emmetropic and highly myopic eyes. This result may be not unexpected because the current OAG eyes were in relatively early stage of the disease (Mean MD was approximately –4.0 dB), and height variation contour has been reported to be less reliable in diagnosing glaucoma. [25], [26] In contrast, a significant decrease in RNFL cross sectional area was observed only in the emmetropic glaucomatous eyes. To the best of our knowledge, this is the first report describing the effect of glaucoma on the sector wise HRT parameters in highly myopic eyes. As in global analysis, rim area was smaller and cup disc area ratio and mean cup depth were larger in OAG eyes in all of the 6 sectors in emmetropic eyes and the most of the 6 sectors in highly myopic eyes. Sectorwise analyses of RNFL cross sectional area also yielded compatible results with its global analysis.

RNFL cross sectional area was significantly smaller in all sectors in emmetropic OAG eyes compared with those in normal eyes, while it was significantly smaller in highly myopic OAG eyes only in the temporal-inferior sector (Table 2). HRT-determined RNFL cross sectional area indicated total cross sectional area of the retinal nerve fiber along the contour line (disc margin) measured relative to the reference plane, which is different from the retinal nerve fiber layer thickness determined by spectral-domain optical coherence tomography along a circle with diameter of approximately 3.4 mm centered on the disc center. In myopic glaucomatous disc with myopic change of the optic nerve head complex in highly myopic eyes [15], [16], [27] positioning of the reference plane relative to the disc structures is expected to different from that in emmetropic eyes. It may be possible that HRT-determined RNFL cross sectional area is less sensitive to the early-stage glaucomatous change in highly myopic eyes.

FSM discriminant function is a HRT II inbuilt linear discriminant function developed by Mikelberg et al.,[22] using rim volume, cup shape measure, and height variation contour in the formula. The RB discriminant function uses rim area, height variation contour, cup shape measure, and RNFL thickness as the best variables to differentiate healthy from glaucomatous eyes. MRA is also a linear discriminant function which accounts for the relationship between optic disc size and rim area or cup-disc area ratio. [3] The sensitivity and specificity of these methods varies according to the study populations. In the original populations, the FSM discriminant function exhibited 0.85 sensitivity and 0.84 specificity [22] and MRA yielded 0.75 sensitivity and 0.98 specificity values. [3].

According to the analyses of ROC curves in emmetropic eyes, there were no significant differences among AUCs of FSM, RB discriminant function, and MRA discriminant function (Figure 1). AUC of each of sole HRT parameter was also calculated. AUCs of rim area, cup/disc area ratio, mean cup depth, height variation contour, cup shape measure, and RNFL cross sectional area were 0.889(95%CI: 0.828–0.950), 0.922(0.858–0.976), 0.864(0.798–0.930), 0.516(0.410–0.622), 0.861(0.794–0.927), and 0.697(0.604–0.790) in the emmetropic eyes, and those were 0.728(0.641–0.815), 0.805(0.730–0.881), 0.765(0.684–0.846), 0.611(0.513–0.709), 0.783(0.705–0.861), 0.527(0.427–0.628) in the highly myopic eyes. Cup/disc area ratio had highest AUCs in both emmetropic and highly myopic eyes, and those were not significantly different from AUCs of FSM, RB discriminant function, or MRA discriminant function (*P*>0.073). AUCs of cup/disc area ratio, RB and MRA discriminant function were significantly smaller in the highly myopic eyes than in emmetropic eyes, while AUC of FSM was not significantly decreased and significantly higher than RB and MRA discriminant function in the highly myopic eyes (Figure 2). These results suggest that FSM algorithm has an advantage in discriminating glaucoma in highly myopic eyes than RB or MRA discriminant function.

In the emmetropic subjects from the current study, sensitivity and specificity of FSM discriminant function and MRA 1 (only “outside normal limits” eyes were diagnosed as glaucomatous) were similar to the above mentioned reports, supporting the validity of the subjects, though RB discriminant function showed relatively lower sensitivity than the other methods. In the highly myopic eyes, sensitivity and specificity of each method were generally smaller than those in the emmetropic eyes. In respect of sensitivity, FSM and MRA 2 (“outside normal limits” and “border line” eyes were diagnosed as glaucomatous) had relatively high sensitivity (0.75 and 0.76) rather than RB discriminant function (0.22) or MRA 1 (0.51).Those methods may be suitable for screening for glaucoma in highly myopic eyes with a smaller beta error (false negative), though specificity of FSM or MRA 2 was lower than that for RB discriminant function and MRA 1. In respect of specificity, on the other hand, RB discriminant function and MRA 1 had relatively high specificity (0.90 and 0.89) than FSM or MRA 2 in the highly myopic eyes. Those methods may be suitable for excluding glaucoma in highly myopic eyes with a small alpha error (false positive), while sensitivities of these methods were low in this population.

The HRT parameters were compared between the eyes with erroneous diagnosis of glaucoma or non-glaucoma and those with correct diagnosis in highly myopic eyes to find possible tendency or features affecting glaucoma diagnosis using HRT in highly myopic eyes. In general, the non-glaucoma eyes with erroneous diagnosis of glaucoma had significantly thinner rim and larger cup and vice versa in glaucomatous eyes with erroneous diagnosis of normal as expected, and there were no significant difference in disc area between them with each of the discriminant method of FSM, RB, MRA 1, or MRA 2 (*P*>0.08 after Bonferroni’s correction). There were no eyes with obvious morphological features, such as large peripapillary atrophy, in those eyes because eyes with pathologic myopic changes had been excluded from the subjects.

A possible caveat of this study is as follows. In the current study, the average age was greater in the emmetropic eyes than in the highly myopic eyes (approximately 60 years of age versus 40 years of age). In non-glaucomatous Japanese subjects, disc area, rim area, mean (or maximum) cup depth, height variation contour, and RNFL cross sectional area had significant negative correlations with age, while cup/disc area ratio and cup shape measure had significant positive correlations with age. [28], [29] Although the parameters have been compared after adjustment for age and disc area in this study, mathematical correction by means of ANCOVA may not be perfect.

One of the most critical drawbacks of the studies using HRT in the morphological analysis of optic disc or peripapillary retina is that positioning of a reference plane relatively to the disc structures is expected to be different between emmetropic eyes and highly myopic eyes with myopia associated optic nerve head complex changes. In any case, this is methodological limitation in morphologically analyzing optic disc using HRT. For this reason, comparison for HRT parameters was limited to between emmetropic normal and OAG eyes or between highly myopic normal and OAG eyes. Since it was tried to include physiologic intermediate myopia which prevails frequently up to relatively high level of myopia (–10D) [27] and to exclude carefully eyes with signs of suggestive of pathologic myopia, it is unlikely that HRT parameters obtained such as rim area in the current highly myopic eyes represented disc structures different from those in emmetropic eyes. Since it is theoretically possible to analyze optic disc morphology after correcting the tilting of the plane of Bruch’s membrane’s opening by spectral domain OCT (SD-OCT), it would be better to investigate the optic disc morphology using SD-OCT in future.

In summary, glaucomatous changes in ONH morphology were investigated based on the HRT parameters of the emmetropic and highly myopic eyes. Rim area, cup/disc area ratio, mean cup depth, and cup shape measure area significantly different from normal eyes both in emmetropic eyes and highly myopic eyes with glaucoma. On the other hand, previously reported thinning of the RNFL cross sectional area in glaucomatous eyes was not encountered in the current highly myopic eyes, which suggested difference in positioning of the reference plane relative to the retinal surface between the current emmetropic and highly myopic eyes and possible difference in the effect of early stage glaucomatous change on this parameter between the two groups. Diagnostic performance of glaucoma using HRT with several discriminant formulas was significantly reduced in highly myopic eyes probably because those eyes were excluded from the original studies which determined those formulas. ROC curve analysis indicated that FSM may have better diagnostic accuracy in discriminating glaucoma in highly myopic eyes. Further, FSM and MRA 2 (“outside normal limits” and “border line” eyes were diagnosed as glaucomatous) diagnosed more glaucomatous eyes with high myopia and RB discriminant function and MRA 1 (only “outside normal limits” eyes were diagnosed as glaucomatous) had relatively high specificity in the highly myopic non-glaucomatous eyes in the subjects of the present study.
